# Evaluating age-based vital sign cutoffs for pediatric trauma: a multicenter evaluation of the Japanese trauma data bank

**DOI:** 10.1007/s00068-025-02985-6

**Published:** 2025-10-28

**Authors:** Mafumi Shinohara, Takeru Abe, Jillian K. Gorski, Sriram Ramgopal

**Affiliations:** 1 Advanced Critical Care and Emergency Center, Department of Emergency Medicine, Yokohama City University Medical Center, Yokohama City, Kanagawa Japan; 2 Center for Integrated Sciences and Humanities, Fukushima Medical University, Fukushima, Japan; 3 Division of Pediatric Emergency Medicine, Department of Emergency Medicine, University of Wisconsin School of Medicine & Public Health, Madison, WI USA; 4 Division of Emergency Medicine, Ann & Robert H. Lurie Children’s Hospital of Chicago, Chicago, IL USA; 5 Department of Pediatrics, Northwestern University Feinberg School of Medicine, Chicago, IL USA; 6 Stanley Manne Children’s Research Institute, Chicago, IL USA; 7 Department of Pediatrics, Division of Pediatric Emergency Medicine, Ann & Robert H. Lurie Children’s Hospital of Chicago, 225 E Chicago Ave, Box 62, Chicago, IL 60611-2991 USA

**Keywords:** Vital signs, Prehospital, Major trauma, Tachypnea, Hypotension

## Abstract

**Purpose:**

To evaluate differing vital signs criteria for the prediction of major trauma in children.

**Methods:**

We conducted a multicenter retrospective cohort study including children (< 18 years) from the 2019–2022 Japan Trauma Data Bank. We compared Pediatric Advanced Life Support (PALS), Advanced Trauma Life Support (ATLS), and empirically-derived criteria for first-measured emergency department vital signs. The primary outcome was major trauma, defined as having an elevated Injury Severity Score and meeting Need for Trauma Intervention criteria (mortality, transfusion, intensive care unit admission, mechanical ventilation, and/or performance of surgery or interventional radiology procedure). We evaluated the diagnostic accuracy of each criterion and their performance in a multivariable logistic regression model.

**Results:**

Of 3,798 children included, 417 (11.0%) had major trauma. For heart rate, all criteria showed similar performance (sensitivity 42.2–45.3%, specificity 75.4–79.2%). For respiratory rate, ATLS had low sensitivity (10.6%, 95% confidence interval [CI] 7.8–13.9%) but high specificity (94.1%, 95% CI 93.2–94.8%). PALS and empirically-derived criteria had moderate sensitivity (~ 60%) and specificity (~ 60%). For systolic blood pressure, PALS had higher sensitivity (68.2%, 95% CI 63.5–72.7%) but lower specificity (42.5%, 95% 37.4–47.1%) than empirically derived cutoffs; ATLS had low sensitivity (12.5%, 95% CI 9.5–16.0%) but very high specificity (98.3%, 95% CI 97.8–98.7%). Multivariable area under the receiver operator characteristic curves were 0.65 for PALS, 0.68 for empirically derived, and 0.63 for ATLS.

**Conclusion:**

PALS and empirically-derived criteria outperformed ATLS in identifying children with major trauma. These findings signal an opportunity to refine vital sign thresholds in pediatric trauma triage.

**Supplementary information:**

The online version contains supplementary material available at 10.1007/s00068-025-02985-6.

## Introduction

Trauma is a leading cause of child death in many high-income countries globally [1, 2]. The early recognition and triage of injured children is an essential to improving outcomes for these patients [3]. Physiological measures, including vital signs, are recognized to have greater importance in trauma triage compared to mechanistic criteria [3], thus representing critical criteria for trauma team activation [4, 5]. Several criteria may be used to classify vital signs as abnormal versus normal based on a child’s age [6], including Pediatric Advanced Life Support (PALS, published by the American Heart Association) [7] and Advanced Trauma Life Support (ATLS, published by the American College of Surgeons) criteria [8].

For injured children, early identification of major trauma- defined as a severe injury that causes physiological instability or death- is essential to determine the need for immediate intervention or intensive care [9]. Recent work has attempted data driven approaches towards the development of vital signs parameters that may be useful in risk stratification of major trauma [10, 11]. In one study, Gorski, et al. empirically derived and internally validated vital sign ranges using the US National Trauma Data Bank (NTDB), and compared these to PALS and ATLS vital signs criteria [11]. These cutoffs were derived using cutpoint analysis on age-normed vital signs to identify upper and lower bounds of vital signs which were maximally associated with the presence of major trauma, defined as having significant injury severity combined with receipt of emergent trauma-relevant interventions. Their findings suggested that empirically derived vital sign criteria effectively balanced the sensitivity of the PALS criteria and the specificity of the ATLS criteria [11]. Comparing PALS and ATLS with empirically derived cutoffs provides essential clinical context and highlights whether newer criteria meaningfully improve upon established standards.

Trauma systems vary significantly between countries and regions. External validation of empirically derived vital sign ranges is a critical next step to ensure their broader applicability and reliability. This is particularly important in distinct clinical settings, where patient populations, injury patterns, and care environments may differ significantly [12]. Our primary objective was to therefore evaluate the distribution of abnormal vital signs among children in a Japanese sample of patients. Second, we sought to evaluate the association of differing criteria of abnormal vital signs for detecting major trauma in this sample.

## Methods

### Data source

We conducted a multicenter, retrospective cohort study utilizing data from the Japan Trauma Data Bank (JTDB), a nationwide trauma registry established in 2004 by the Japanese Association for the Surgery of Trauma and the Japanese Association for Acute Medicine. The purpose of the JTDB is to improve and maintain the quality of trauma care in Japan. The registry collects data on patient and hospital information, including demographics, premorbid medical conditions, vital signs, Abbreviated Injury Scale scores, Injury Severity Scores (ISS), in-hospital procedures, and in-hospital and emergency department (ED) mortality. For the present study, we analyzed JTDB data from 2019 to 2022, including data from 303 hospitals. The study was approved by the Yokohama City University Institutional Review Board (F230300003) with a waiver of informed consent. This study adhered to the Strengthening the Reporting of Observational Studies in Epidemiology guidelines for reporting observational research [13].

### Inclusion

We included all trauma encounters for children (defined as age < 18 years) from the study years. We excluded encounters for children who presented in cardiac arrest (defined as receiving cardiopulmonary resuscitation upon ED arrival and prehospital), encounters for burns, encounters following interfacility transport, and encounters with missing documentation of all vital signs.

### Data extraction

We extracted the following data from the study dataset: age, sex, injury mechanism, injury classification (blunt, penetrating, or combined), and data required to calculate in-hospital outcomes. From data available within the JTDB, we classified injury mechanism as traffic accident, fall, machinery injury, struck by or against, pressure, or other.

### Exposures

Our primary exposure of interest was the first-documented ED vital signs, including heart rate (HR), respiratory rate (RR), and systolic blood pressure (SBP). We classified HR, RR, and SBP based on three age-specific criteria: 2020 PALS [7] 2018 ATLS [8], and empirically-derived criteria first described in Gorski, et al. [11]. The ATLS criteria provide upper HR and RR limits and a lower SBP limit, whereas the PALS and empirically derived criteria provide both upper and lower limits for each vital sign. These vital signs criteria are summarized in Supplementary Table 1.

### Outcome

Our primary outcome was a composite measure derived from available clinical outcomes within the JTDB, consistent with the outcome of major trauma used by Gorski, et al. [11]. This outcome used a modified Standard Triage Assessment Tool (STAT) outcome [14], defined as having an elevated injury severity (ISS >15) score and meeting the Need for Trauma Intervention (NFTI) criteria [15]. The NFTI criteria are defined as the presence of one of the following: (1) in-hospital mortality within 60 h, (2) transfusion of packed red blood cells within 4 h of hospital arrival, (3) intensive care unit hospitalization for ≥ 3 days, (4) use of mechanical ventilation within 3 days of hospital arrival, (5)initiation of surgery within 90 min of ED arrival, (6) initiation of interventional radiology within 90 min of ED arrival [15]. The time intervals used in NFTI definitions were developed by the authors of these criteria to reflect the temporal progression of injuries and their associated interventions, with the goal of identifying injuries requiring intervention [16].

### Analysis

We described our sample, stratified by the presence or absence of major trauma. Among encounters with each available vital sign, we calculated diagnostic accuracy measures for major trauma, including sensitivity, specificity, positive predictive value (PPV), negative predictive value (NPV), positive likelihood ratio, and negative likelihood ratio. All measures were reported with corresponding 95% confidence intervals (CIs). Next, we performed logistic regression analysis to evaluate the association of the number of abnormal vital signs (ranging from 0 to 3) with the presence of major trauma. We calculated the area under the receiver operating characteristic curve (AUROC), with 95% CI. We used the DeLong method to compare AUROCs generated from the three vital sign criteria to assess significant differences. A two-sided *p* < 0.05 was considered statistically significant. From each receiver operating characteristic curve, we calculated the optimal cutpoint using the Youden Index and reported the sensitivity and specificity for each. We evaluated characteristics of patients with missing vital signs who were excluded from the primary analysis. As an *exploratory analysis*, we repeated these steps for each component used to define major trauma as each of the following: (1) either elevated ISS (> 15) or NFTI criteria, (2) an elevated ISS (> 15), and (3) the NFTI criteria. Additionally, we repeated our analysis on the subset of children < 14 years of age, which has been used as a threshold for pediatric trauma in other work.

Analyses were performed using STATA software (Stata/SE 13.0, StataCorp LLC, TX, USA) and R, version 4.3.2 (R Foundation for Statistical Computing, Vienna, Austria).

## Results

### Inclusion

**Fig. 1:**
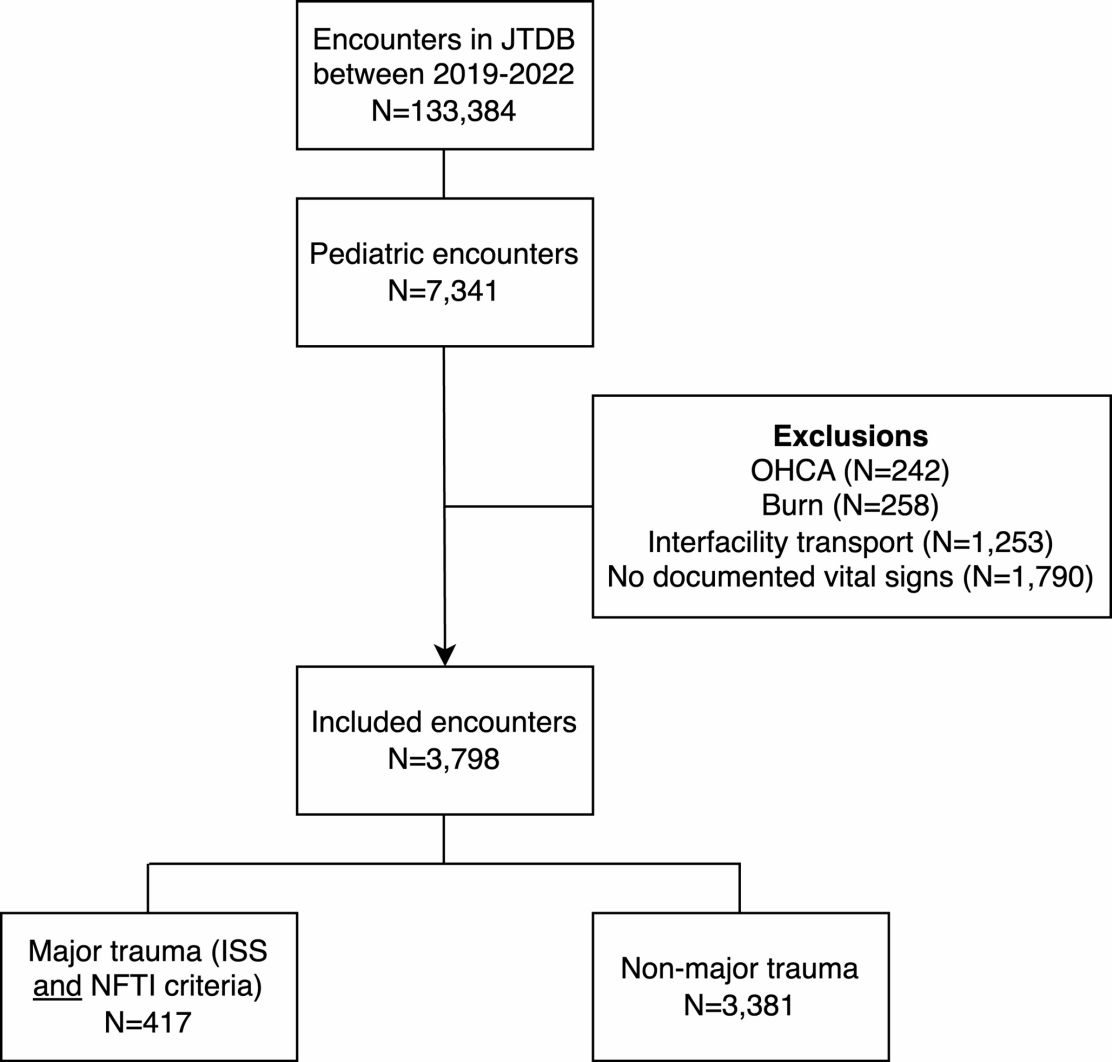
Patient inclusion JTDB, Japan Trauma Data Bank; OHCA, out of hospital cardiac arrest; NFTI, Need for Trauma Intervention, ISS, injury severity score

A total of 133,384 encounters were recorded in the JTDB during the study period. Among these, 7,341 (5.5%) involved children. After applying exclusion criteria, 3,798 encounters were retained for analysis (Fig. 1). Most included encounters were for males (69.0%). The median encounter age was 13 years (IQR 8–16). A minority of events (1.8%) were for penetrating trauma. Demographic characteristics are presented in Table 1.

**Table 1:** Sample demographics. Numbers in cells represent median [IQR] or N (%)

Variable	All patients	Major trauma	No major trauma
Number	3,798	417	3,381
Age, years	13 [8–16]	14 [9–16]	12 [8–16]
Sex			
Male	2,619 (69.0)	268 (64.3)	2,351 (69.5)
Female	1,143 (30.1)	146 (35.0)	997 (29.5)
Missing	36 (0.9)	3 (0.7)	33 (1.0)
Type of injury			
Penetrating	68 (1.8)	6 (1.4)	62 (1.8)
Blunt	3,677 (96.8)	406 (97.4)	3,271 (96.7)
Both penetrating and blunt	6 (0.2)	0 (0)	6 (0.2)
Unknown	47 (1.2)	5 (1.2)	42 (1.2)
Mechanism			
Traffic related	2,099 (55.3)	249 (59.7)	1,850 (54.7)
Fall	1,261 (33.2)	145 (34.8)	1,116 (33.0)
Machinery	14 (0.4)	1 (0.2)	13 (0.4)
Struck by or against	325 (8.6)	18 (4.3)	307 (9.1)
Pressure	15 (0.4)	1 (0.2)	14 (0.4)
Other	60 (1.6)	2 (0.2)	58 (1.7)
Missing	24 (0.6)	1 (0.2)	23 (0.7)
Emergency department disposition			
Hospital admission	3,668 (96.6)	399 (95.7)	3,269 (96.7)
Intensive care unit	2,292 (60.3)	387 (92.8)	1,905 (56.3)
Ward	1,284 (33.8)	6 (1.4)	1,278 (37.8)
Unknown	92 (2.4)	6 (1.4)	86 (2.5)
Discharge	24 (0.6)	0 (0)	24 (0.7)
Transfer	74 (1.9)	10 (2.4)	64 (1.9)
Death	7 (0.2)	7 (1.7)	0 (0)
Unknown/Missing	25 (0.7)	1 (0.2)	24 (0.7)
ISS	9 [4–16]	25 [19–33]	9 [4–12]
ISS > 15	922 (24.3)	417 (100)	505 (14.9)
ISS > 25	279 (7.3)	187 (44.8)	92 (2.7)
NFTI	646 (17.0)	417 (100)	229 (6.8)

*ICU*; intensive care unit, *ISS*; injury severity score, *NFTI*; Need for Trauma Intervention

### Study outcomes

Overall, 922 (24.3%) children had an ISS > 15, and 646 (17.0%) children received at least one of the NFTI interventions. The number of patients in each intervention was as follows (1) in-hospital mortality within 60 h: *n* = 30, (2) transfusion of packed red blood cells within 4 h of hospital arrival: *n* = 136, (3) intensive care unit hospitalization for ≥ 3 days: *n* = 550, (4) use of mechanical ventilation within 3 days of hospital arrival: *n* = 211, (5) initiation of surgery within 90 min of ED arrival: *n* = 56, (6) initiation of interventional radiology within 90 min of ED arrival: *n* = 37. The composite outcome, defined as meeting both criteria, occurred in 417 cases (11.0%).

### Vital sign abnormalities by criterion

**Fig. 2:**
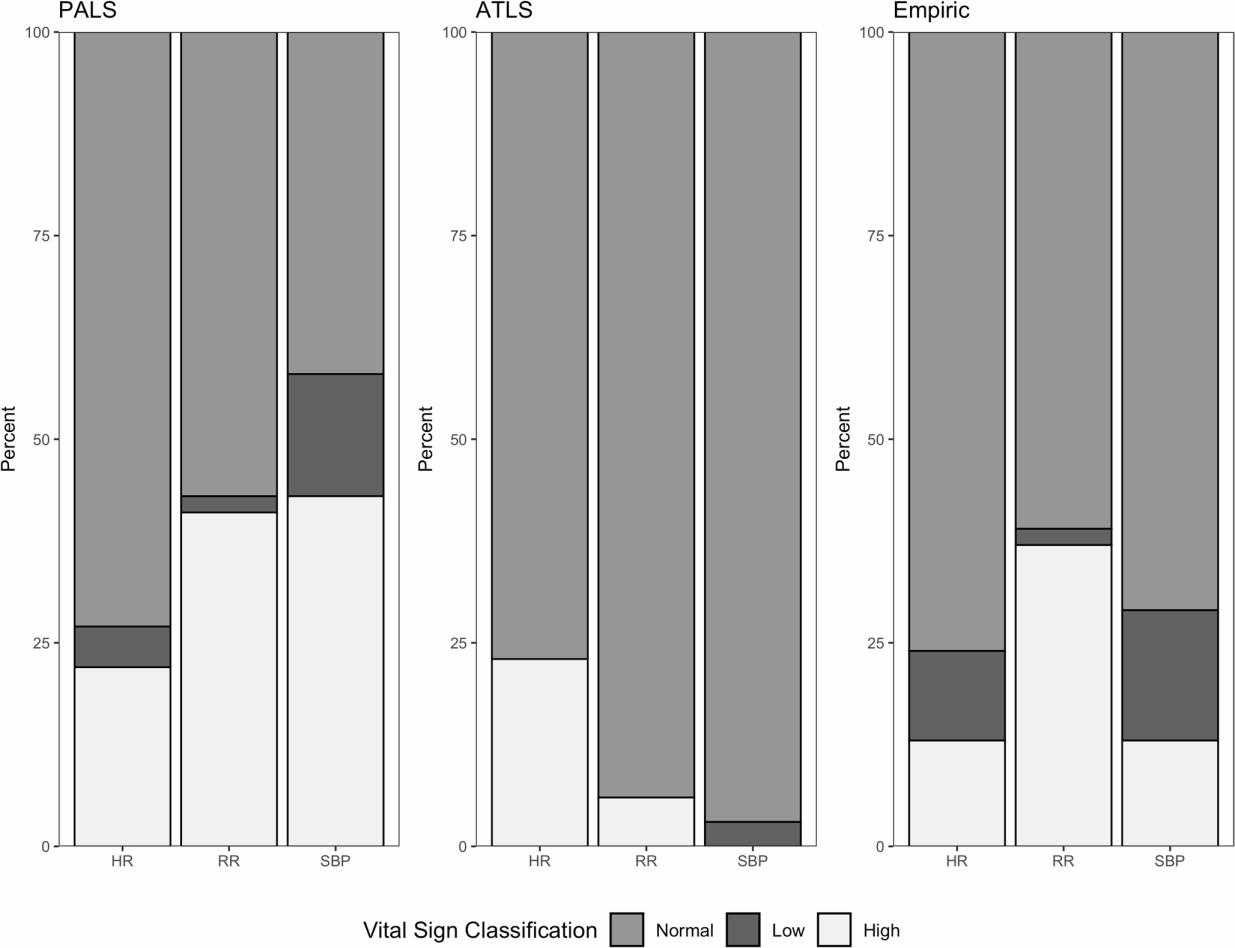
Distribution of normal versus abnormal vital signs using each criterion

The PALS criteria identified the highest percentage of patients with abnormal vital signs, followed by the empirically derived criteria (Fig. 2). The ATLS criteria classified the highest percentage of vital signs as normal, with 77% of HR, 94% of RR, and 97% of SBP considered as normal.

### Application of previously developed model to predict major trauma

Measures of diagnostic accuracy for each vital sign criterion with corresponding 95% CI are provided in Table 2. For HR, PALS, ATLS and empirically-derived cutoffs had similar measures of performance, with sensitivities ranging from 42.2 to 45.3% and specificities ranging from 75.4 to 79.2%. For RR, empirically-derived and PALS criteria had similar sensitivities (60.2% for PALS, 57.6% for empirically-derived criteria) and specificity (59.1% for PALS, 62.6% for empirically-derived criteria). ATLS criteria, in contrast, demonstrated comparatively low sensitivity (10.6%) and high specificity (94.1%). For SBP, PALS had higher sensitivity (68.2%) compared to empirically-derived criteria (47.7%) but lower specificity (42.5% for PALS, 72.7% for SBP). ATLS criteria demonstrated low sensitivity (12.5%) and extremely high specificity (98.3%). Among 731 with normal vital signs when using PALS criteria, 30 (4.1%) had major trauma. Similarly, among 1340 patients with normal vital signs using empirically derived criteria, 55 (4.1%) had major trauma. In contrast, major trauma occurred more frequently (213 [7.8%]) among encounters classified as having normal vital signs when using ATLS criteria (*n* = 2737).

**Table 2:** Comparison of empirically derived vital sign criteria, the PALS criteria, and the ATLS criteria for detecting major trauma. The prevalence of major trauma was 11.0%

Vital sign criteria	Sensitivity, % (95% CI)	Specificity, % (95% CI)	PPV, % (95% CI)	NPV, % (95% CI)	PLR (95% CI)	NLR (95% CI)
PALS						
HR	45.3 (40.4–50.1)	75.4 (73.9–76.8)	18.5 (16.1–21.0)	91.8 (90.7–92.8)	1.84 (1.63–2.08)	0.73 (0.66–0.79)
RR	60.2 (55.3–64.9)	59.1 (57.4–60.8)	15.4 (13.6–17.2)	92.3 (91.1–93.4)	1.47 (1.35–1.61)	0.67 (0.60–0.76)
SBP	68.2 (63.5–72.7)	42.5 (40.8–44.2)	12.7 (11.4–14.2)	91.6 (90.1–92.9)	1.19 (1.10–1.27)	0.75 (0.65–0.87)
ATLS						
HR	42.2 (37.4–47.1)	79.1 (77.7–80.5)	20.0 (17.4–22.8)	91.7 (90.7–92.7)	2.02 (1.78–2.31)	0.73 (0.67–0.79)
RR	10.6 (7.8–13.9)	94.1 (93.2–94.8)	18.0 (13.4–23.3)	89.5 (88.4–90.5)	1.77 (1.30–2.42)	0.95 (0.92–0.98)
SBP	12.5 (9.5–16.0)	98.3 (97.8–98.7)	47.3 (37.7–57.0)	90.1 (89.1–91.0)	7.27 (5.07–10.42)	0.89 (0.86–0.92)
Empirically derived using simplified age-based cutoff						
HR	44.3 (39.5–49.3)	78.1 (76.7–79.5)	20.0 (17.4–22.7)	91.9 (90.9–92.9)	2.03 (1.79–2.30)	0.71 (0.65–0.78)
RR	57.6 (52.7–62.4)	62.6 (61.0–64.3.0.3)	15.9 (14.1–17.9)	92.3 (91.1–93.4)	1.54 (1.40–1.69)	0.68 (0.60–0.76)
SBP	47.7 (42.8–52.6)	72.7 (71.1–74.2)	17.7 (15.5–20.0)	91.9 (90.8–92.9)	1.74 (1.56–1.96)	0.72 (0.65–0.79)

*ISS*; Injury Severity Score, *NFTI*; Need for Trauma Intervention, *HR*; heart rate, *RR*; respiratory rate, *SBP*; systolic blood pressure

### AUROC of each vital sign criteria

**Fig. 3:**
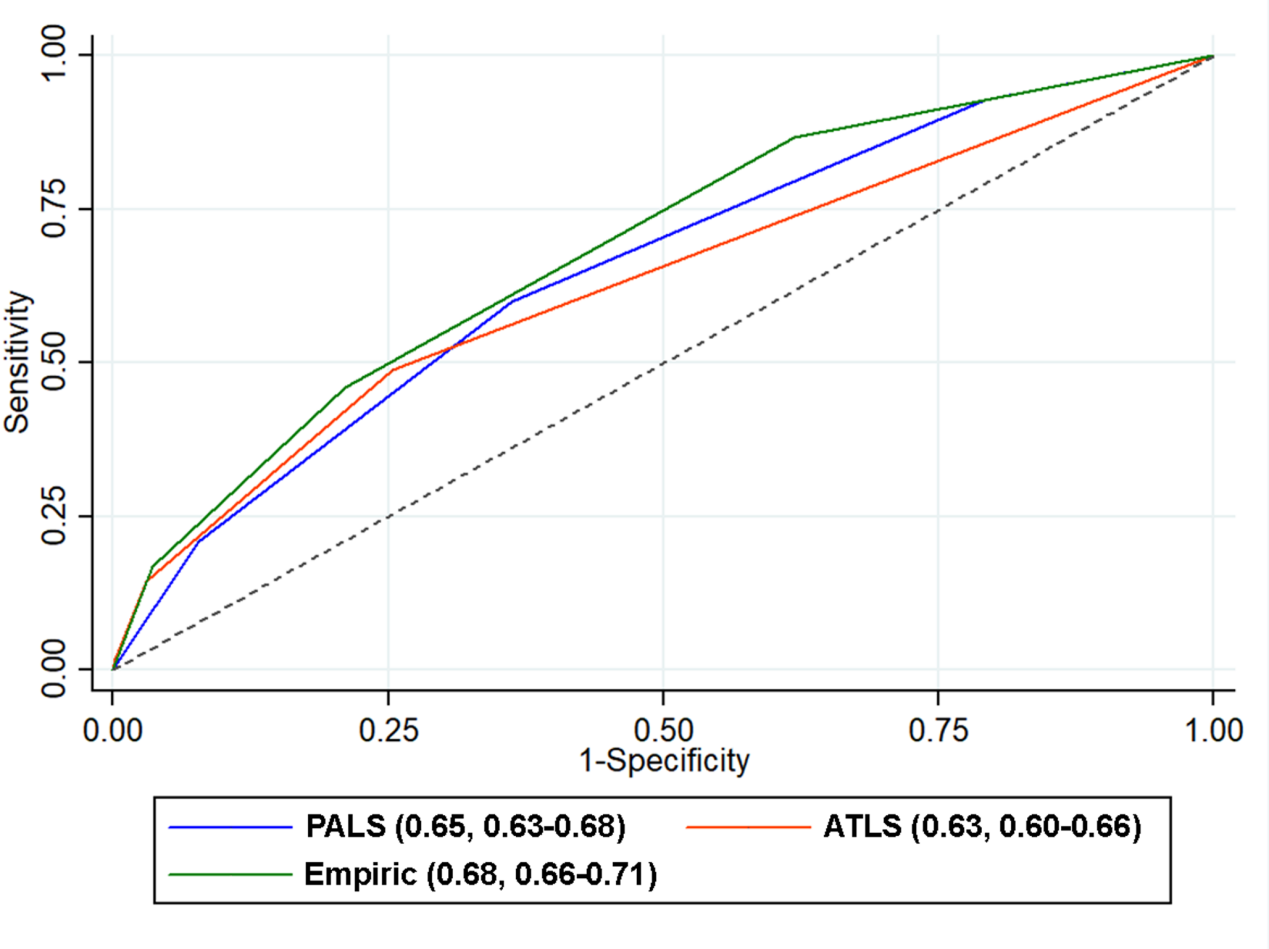
Area under the receiver operator characteristic curves for each vital signs criteria following logistic regression using an outcome of major trauma

The AUROCs with 95% CI for multivariable models using each vital sign criterion are shown in Table 3. The AUROCs for models using the PALS and ATLS criteria were similar (0.65 [95% CI; 0.63–0.68] and 0.63 [95% CI; 0.60–0.66] respectively; *p* = 0.06. The AUROC for empirically derived criteria (0.68, 95% CI 0.66–0.71) was significantly higher than that of the PALS criteria and ATLS criteria (*p* = 0.002 and 0.0001, respectively; Fig. 3). At optimally selected cutpoints (1.5 points for PALS and empiric criteria; 0.5 points for ATLS criteria), the sensitivity of the PALS was higher (0.60, 95% CI 0.55–0.64) than for empiric criteria (0.46, 95% CI 0.41–0.51) and ATLS criteria (0.49, 95% CI 0.44–0.54).

**Table 3:** The area under the receiver operating characteristic curve (AUROC) with 95% confidence intervals (CI) for major trauma

Criterion	AUROC	95% CI	Sensitivity	Specificity
PALS	0.65	0.63–0.68	0.60 (0.55–0.64)	0.64 (0.62–0.66)
ATLS	0.63	0.60–0.66	0.49 (0.44–0.54)	0.75 (0.73–0.76)
Empirically derived ranges	0.68	0.66–0.71	0.46 (0.41–0.51)	0.79 (0.77–0.80)

*PALS*; Pediatric Advanced Life Support, *ATLS*; Advanced Trauma Life Support

### Missing vital signs

Among 1,790 children with missing vital signs, major trauma occurred in a lower percentage of encounters (7.9%). Compared to included patients with vital signs data, patients with missing vital signs were frequently younger (median age 7 years compared to 12 years), more frequently had a traffic-related mechanism of injury, and had slightly lower mortality. Characteristics of this sample are provided in Supplementary Table 2.

### Evaluation of alternative outcomes

When major trauma was defined as meeting either the ISS > 15 or NFTI criteria, a total of 1,151 (30.3%) patients were identified as having major trauma. Compared to when it was defined as meeting both the ISS > 15 and NFTI criteria, the numerical trend was similar to the primary analysis (Supplementary Table 3). Results for outcomes using based on the ISS > 15 criterion alone (*n* = 922 patients) and the NFTI criterion alone (*n* = 646 patients) are presented in Supplementary Tables 4 and 5. In both cases, the results were again comparable to the primary analysis.

### Children < 14 years of age

When limiting our analysis to younger children, 2,302 children were included, of which 213 had major trauma (9.3%). Findings within this subgroup were comparable to those in the primary analysis (Supplementary Table 6).

## Discussion

We used the JTDB to evaluate the association of vital signs with major trauma in children presenting to Japanese trauma centers. We found that empirically-derived vital sign criteria had greater sensitivity for identifying major trauma than the ATLS criteria and greater specificity than the PALS criteria. The empirically-derived and PALS criteria had similar performance when incorporated into a multivariable model, both of which exceeded ATLS criteria. These results validate findings described by Gorski, et al., in their derivation of vital sign ranges for injured children and can be used to inform approaches used in the risk stratification of injured children.

We identified several similarities between our findings using two disparate trauma systems. We found that the distribution of vital sign abnormalities using the PALS, ATLS and empirically-derived criteria was similar between the US sample (as described by Gorski, et al.), and the present sample from the JTDB [11]. We found a higher percentage of children meeting major trauma criteria in the JTDB sample (11.0%, versus 4.6% from the NTDB), which appears to be driven by a higher proportion of children with elevated ISS in the JTDB sample. This may be due to differences in data sources, as the JTDB has a higher proportion of Designated Trauma Centers and Emergency and Critical Care Centers, which serve a higher acuity sampling of patients, and with fewer community hospitals and general EDs, which are more frequently represented in the NTDB [17]. The JTDB primarily includes cases with an ISS >3 (though some cases with ISS ≤ 3 are registered), which may have influenced the results [17]. Nevertheless, we found that most measures of diagnostic accuracy were similar to those reported from the JTDB for all three vital signs criteria.

By deriving vital signs based on the presence or absence of major trauma, the use of empirically-derived vital sign ranges allows for improved prehospital and early triage. This in turn may improve the development of timely decisions regarding trauma team activation and other interventions. Prior work has highlighted high rates of under-triage in children, with several vital-sign related factors associated with the occurrence of this event [18]. Existing field triage criteria for injured children in the U.S. include abnormal RR (not adjusted for age), hypotension in children under 10 years (defined as systolic blood pressure < 70 mmHg + 2 × age in years), and shock index for children aged 10 years and older [3]. One systematic review which included 5 eligible observational studies conducted in the U.S. between 1996 and 2017 reported that the sensitivity of field triage protocols for pediatric trauma ranged from 49.1% to 87.3% and specificity from 41.7% to 84.8%, with no protocol meeting the international benchmark of 95% sensitivity [19]. While these findings are specific to US-systems, they likely generalize to other industrialized healthcare systems. When using vital signs as a triage tool for major trauma, minimizing under-triage is considered most critical; conversely, some degree of over-triage is generally acceptable to ensure patient safety. Similar to Gorski, et al., we found that ATLS criteria have low sensitivity for major trauma, which may increase the risk of missing major trauma when incorporated into a field trauma decision algorithm [18]. In contrast, the empirically-derived and PALS criteria demonstrated substantial improvements in these measures. Other studies, including one reported by in a metropolitan Japanese region, have noted that using empirically-derived vital sign ranges in children improve the overall identification of children at higher risk of requiring critical lifesaving procedures or have in-hospital critical outcomes, compared to existing vital sign ranges [20–22].

Our findings offer meaningful implications to improve clinical decision-making and trauma system protocols in both prehospital and hospital settings. The integration of empirically-derived criteria into trauma protocols, especially within systems of varying hospital capability and pediatric expertise, can improve the identification of children at higher risk of having major trauma. Importantly, given that the empirically-derived criteria were developed using U.S.-based data but performed comparably in a Japanese context, their generalizability across diverse trauma systems appears promising. This cross-validation may also facilitate future multinational consensus on vital sign thresholds, ultimately contributing to more harmonized and evidence-based pediatric trauma triage guidelines. Our findings evaluating age-based vital signs criteria parallel other work using data-driven approaches to derive and evaluate age-based cutoffs for the shock index in children, using clinically important outcomes [23, 24].

Our findings have several limitations. This was a retrospective analysis of an existing database, which may contain errors in registration and coding. Some vital signs were incomplete, leading to an exclusion of cases. The reasons for these missing data are unclear but may include circumstances in which measurement was not possible, failure to measure, failure to record measured data, or omissions during data entry. Although the JTDB is a nationwide database, it does not include all injured and transported patients. In particular, patients with mild trauma (ISS ≤ 3) may be underrepresented, introducing potential selection bias. As this was a retrospective observational study, causality between vital signs and outcomes could not be established. Despite these limitations, these findings provide a useful external validation of vital sign ranges and support continued research evaluating their use and integration within trauma systems.

## Conclusion

In this study evaluating a multicenter cohort of injured children in Japan, we found that empirically-derived vital sign criteria demonstrated greater sensitivity than the ATLS criteria and greater specificity than the PALS criteria. At an optimal cutpoint, the performance of the empirically-derived criteria was similar to that of the PALS criteria and higher than that of the ATLS criteria. Using a distinct sample, our findings confirm that ATLS criteria are poorly sensitive for major trauma, while empirically derived and PALS criteria offer more balanced, though differing, tradeoffs between sensitivity and specificity. Future efforts should combine these criteria with additional factors to develop enhanced triage tools.

## Supplementary information

Below is the link to the electronic supplementary material.

ESM 1 (DOCX 51.7 KB)

## Data Availability

The data used within this manuscript is the property of the Japanese Association for Surgery and Trauma (Trauma Surgery Committee) and the Japanese Association for Acute Medicine (Committee for Clinical Care Evaluation).

## Declarations

Approved by the Yokohama City University Institutional Review Board (F230300003).
