# Supplementary material for: Evaluating age-based vital sign cutoffs for pediatric trauma: a multicenter evaluation of the Japanese trauma data bank
Source: Eur J Trauma Emerg Surg. 2025 Oct 28;51(1):323. doi: 10.1007/s00068-025-02985-6 (PMC12568833; doi:10.1007/s00068-025-02985-6)
Supplement: Supplementary file 1 — (DOCX 51.7 KB) [file 68_2025_2985_MOESM1_ESM.docx]

**Supplementary Table 1**. Normal vital sign parameters

|  | | | |
| --- | --- | --- | --- |
| **Age** | **Heart rate (beats/minute)** | **Respiratory rate (breaths/minute)** | **Systolic blood pressure (mmHg)** |
| **Pediatric Advanced Life Support** | | | |
| 0 hours – <12 hours | 100–205 | 30–53 | 39–59 |
| 12 hours – <3 days | 100–205 | 30–53 | 60–76 |
| 3 days – <1 month | 100–205 | 30–53 | 67–84 |
| Infant (1–12 months) | 100–180 | 30–53 | 72–104 |
| Toddler (1–<3 yrs) | 98–140 | 22–37 | 86–106 |
| Preschool (3–<6 yrs) | 80–120 | 20–28 | 89–112 |
| School-age (6–<10 yrs) | 75–118 | 18–25 | 97–115 |
| Preadolescent (10–<12 yrs) | 60–100 | 60–100 | 102–120 |
| Adolescent (12–<19 yrs) | 60–100 | 60–100 | 110–131 |
| **Advanced Trauma Life Support** | | | |
| <1 year | <160 | <60 | >60 |
| 1–2 years | <150 | <40 | >70 |
| 3–5 years | <140 | <35 | >75 |
| 6–12 years | <120 | <30 | >80 |
| 13–18 years | <100 | <30 | >90 |
| **Empiric Vital Signs** | | | |
| 0 months to <3 months | 101-163 | 22-51 | 74-140 |
| 3 months to <6 months | 109-160 | 23-46 | 81-127 |
| 6 months to <9 months | 107-156 | 22-42 | 84-129 |
| 9 months to <1 year | 105-156 | 22-40 | 86-131 |
| 1 year to <3 years | 98-152 | 21-36 | 90-134 |
| 3 years to <6 years | 87-133 | 18-30 | 94-131 |
| 6 years to <9 years | 80-122 | 17-26 | 99-133 |
| 9 years to <12 years | 77-117 | 16-24 | 103-138 |
| 12 years to <18 years | 71-113 | 15-22 | 110-148 |

**Supplementary Table 2.** Characteristics of patients with missing vital signs data and thus excluded from the primary analysis. Numbers in cells represent median (IQR) or N (%)

| Variable | **Missing at least one vital sign** | **All vital signs present** |
| --- | --- | --- |
| Number | 1,790 | 3,798 |
| Age, years | 7 [3-13] | 13 [8-16] |
| Sex |  |  |
| Male | 1,121 (62.6) | 2,619 (69.0) |
| Female | 614 (34.3) | 1,143 (30.1) |
| Missing | 55 (3.1) | 36 (0.9) |
| Type of injury |  |  |
| Penetrating | 40 (2.2) | 68 (1.8) |
| Blunt | 1,618 (90.4) | 3,677 (96.8) |
| Both penetrating and blunt | 2 (0.1) | 6 (0.2) |
| Unknown | 30 (1.7) | 47 (1.2) |
| Mechanism |  |  |
| Traffic related | 445 (24.9) | 2,099 (55.3) |
| Fall | 941 (52.6) | 1,261 (33.2) |
| Machinery | 7 (0.4) | 14 (0.4) |
| Struck by or against | 255 (14.2) | 325 (8.6) |
| Pressure | 10 (0.6) | 15 (0.4) |
| Other | 36 (2.0) | 60 (1.6) |
| Missing | 96 (5.4) | 24 (0.6) |
| Emergency department disposition | | |
| Hospital admission | 1,646 (92.0) | 3,668 (96.6) |
| ICU | 635 (35.5) | 2,292 (60.3) |
| Ward | 951 (53.1) | 1,284 (33.8) |
| Unknown | 60 (3.4) | 92 (2.4) |
| Discharge | 20 (1.1) | 24 (0.6) |
| Transfer | 31 (1.7) | 74 (1.9) |
| Death | 12 (0.7) | 7 (0.2) |
| Unknown/Missing | 81 (4.5) | 25 (0.7) |
| ISS | 9 [4-10] | 9 [4-16] |
| ISS >15 | 344 (19.2) | 922 (24.3) |
| ISS > 25 | 113 (6.3) | 279 (7.3) |
| NFTI | 211 (11.8) | 646 (17.0) |

ICU, intensive care unit; ISS, injury severity score; NFTI, Need for Trauma Intervention

**Supplementary Table 3.** Comparison of empirically derived vital sign criteria, the PALS criteria, and the ATLS criteria for detecting major trauma, define as meeting having an Injury Severity Score>15 and/or meeting the Need for Trauma Intervention criteria.

| **Vital sign criteria** | **Sensitivity, % (95% CI)** | **Specificity, % (95% CI)** | **PPV, % (95% CI)** | **NPV, % (95% CI)** | **PLR (95% CI)** | **NLR (95% CI)** |
| --- | --- | --- | --- | --- | --- | --- |
| **PALS** | | | | | | |
| HR | 36.2 (33.4-39.0) | 77.2 (75.5-78.7) | 40.8 (37.7-43.9) | 73.6 (71.9-75.2) | 1.58 (1.43-1.76) | 0.83 (0.79-0.87) |
| RR | 52.9 (50.0-55.8) | 61.3 (59.4-63.1) | 37.3 (34.9-39.7) | 75.0 (73.1-76.8) | 1.37 (1.27-1.47) | 0.77 (0.72-0.82) |
| SBP | 61.9 (59.0-64.7) | 42.7 (40.8-44.6) | 31.9 (30.0-33.9) | 72.1 (69.8-74.3) | 1.08 (1.02-1.14) | 0.89 (0.82-0.97) |
| **ATLS** | | | | | | |
| HR | 33.4 (30.6-36.2) | 81.2 (79.7-82.7) | 43.6 (40.3-46.9) | 73.7 (72.1-75.3) | 1.78 (1.59-1.99) | 0.82 (0.78-0.86) |
| RR | 8.3 (6.8-10.1) | 94.4 (93.4-95.2) | 39.2 (33.0-45.6) | 70.3 (68.8-71.8) | 1.48 (1.16-1.90) | 0.97 (0.95-0.99) |
| SBP | 7.5 (6.0-9.1) | 99.1 (98.7-99.4) | 78.2 (69.3-85.5) | 71.1 (69.6-72.6) | 8.24 (5.27-12.89) | 0.93 (0.92-0.95) |
| **Empirically derived using simplified age-based cutoff** | | | | | | |
| HR | 33.6 (30.8-36.4) | 79.7 (78.1-81.2) | 41.8 (38.6-45.1) | 73.4 (71.8-75.0) | 1.65 (1.48-1.85) | 0.83 (0.80-0.87) |
| RR | 50.8 (47.9-53.8) | 65.3 (63.5-67.1) | 38.9 (36.4-41.4) | 75.3 (73.5-77.1) | 1.46 (1.36-1.58) | 0.75 (0.71-0.80) |
| SBP | 36.1 (33.3-39.0) | 73.3 (71.5-74.9) | 37.0 (34.1-39.9) | 72.5 (70.8-74.2) | 1.35 (1.22-1.49) | 0.87 (0.83-0.92) |

ISS, Injury Severity Score; NFTI, Need for Trauma Intervention; HR, heart rate; RR, respiratory rate; SBP, systolic blood pressure; CI, confidence interval

**Supplementary Table 4.** Comparison of empirically derived vital sign criteria, the PALS criteria, and the ATLS criteria for detecting major trauma, defined solely based on the NFTI criteria.

| **Vital sign criteria** | **Sensitivity, % (95% CI)** | **Specificity, % (95% CI)** | **PPV, % (95% CI)** | **NPV, % (95% CI)** | **PLR (95% CI)** | **NLR (95% CI)** |
| --- | --- | --- | --- | --- | --- | --- |
| **PALS** | | | | | | |
| HR | 39.1 (35.3-43.0) | 75.6 (74.1-77.1) | 24.8 (22.1-27.5) | 85.8 (84.5-87.1) | 1.61 (1.43-1.80) | 0.80 (0.75-0.86) |
| RR | 56.8 (52.9-60.7) | 59.8 (58.1-61.5) | 22.5 (20.5-24.6) | 87.1 (85.6-88.5) | 1.41 (1.31-1.53) | 0.72 (0.66-0.79) |
| SBP | 64.1 (60.3-67.8) | 42.4 (40.7-44.2) | 18.6 (17.0-20.3) | 85.2 (83.4-87.0) | 1.11 (1.04-1.19) | 0.85 (0.76-0.95) |
| **ATLS** | | | | | | |
| HR | 36.4 (32.7-40.2) | 79.5 (78.1-80.9) | 26.7 (23.8-29.7) | 85.9 (84.6-87.2) | 1.77 (1.57-2.01) | 0.80 (0.75-0.85) |
| RR | 9.1 (7.0-11.6) | 94.1 (93.2-94.9) | 24.1 (18.9-29.9) | 83.5 (82.2-84.7) | 1.55 (1.17-2.05) | 0.97 (0.94-0.99) |
| SBP | 9.8 (7.6-12.3) | 98.5 (98.0-98.9) | 57.3 (47.5-66.7) | 84.2 (83.0-85.4) | 6.54 (4.53-9.45) | 0.92 (0.89-0.94) |
| **Empirically derived using simplified age-based cutoff** | | | | | | |
| HR | 36.5 (32.8-40.3) | 78.2 (76.7-79.6) | 25.5 (22.7-28.5) | 85.7 (84.4-87.0) | 1.67 (1.48-1.89) | 0.81 (0.76-0.86) |
| RR | 53.7 (49.8-57.6) | 63.3 (61.6-65.0) | 23.1 (21.0-25.3) | 87.0 (85.5-88.3) | 1.46 (1.35-1.59) | 0.73 (0.67-0.80) |
| SBP | 40.7 (36.9-44.6) | 72.7 (71.1-74.3) | 23.4 (20.9-26.0) | 85.7 (84.3-87.0) | 1.49 (1.34-1.66) | 0.82 (0.76-0.87) |

ISS, Injury Severity Score; NFTI, Need for Trauma Intervention; HR, heart rate; RR, respiratory rate; SBP, systolic blood pressure; CI, confidence interval

**Supplementary Table 5.** Comparison of empirically derived vital sign criteria, the PALS criteria, and the ATLS criteria for detecting major trauma, defined solely based on an elevated Injury Severity Score (ISS>15).

| **Vital sign criteria** | **Sensitivity, % (95% CI)** | **Specificity, % (95% CI)** | **PPV, % (95% CI)** | **NPV, % (95% CI)** | **PLR (95% CI)** | **NLR (95% CI)** |
| --- | --- | --- | --- | --- | --- | --- |
| **PALS** | | | | | | |
| HR | 38.2 (35.1-41.5) | 76.8 (75.2-78.3) | 34.5 (31.6-37.5) | 79.5 (78.0-81.0) | 1.64 (1.48-1.83) | 0.80 (0.76-0.85) |
| RR | 53.5 (50.2-56.7) | 60.3 (58.5-62.1) | 30.2 (28.0-32.5) | 80.2 (78.4-81.8) | 1.35 (1.25-1.45) | 0.77 (0.72-0.83) |
| SBP | 63.2 (60.0-66.3) | 42.7 (40.9-44.6) | 26.1 (24.3-28.0) | 78.4 (76.3-80.4) | 1.10 (1.04-1.17) | 0.86 (0.78-0.96) |
| **ATLS** | | | | | | |
| HR | 35.2 (32.2-38.4) | 80.7 (79.2-82.1) | 36.9 (33.7-40.2) | 79.5 (78.0-81.0) | 1.82 (1.63-2.05) | 0.80 (0.76-0.84) |
| RR | 8.8 (7.0-10.8) | 94.3 (93.4-95.1) | 33.1 (27.2-39.3) | 76.3 (74.9-77.7) | 1.54 (1.19-1.99) | 0.97 (0.95-0.99) |
| SBP | 8.1 (6.5-10.1) | 98.8 (98.3-99.2) | 68.2 (58.6-76.7) | 77.0 (75.6-78.4) | 6.68 (4.51-9.92) | 0.93 (0.91-0.95) |
| **Empirically derived using simplified age-based cutoff** | | | | | | |
| HR | 36.4 (33.3-39.6) | 79.5 (78.0-81.0) | 36.3 (33.2-39.5) | 79.6 (78.0-81.1) | 1.78 (1.59-1.99) | 0.80 (0.76-0.84) |
| RR | 51.9 (48.6-55.1) | 64.3 (62.6-66.1) | 31.8 (29.4-34.2) | 80.7 (79.0-82.3) | 1.45 (1.34-1.57) | 0.75 (0.70-0.80) |
| SBP | 38.1 (35.0-41.4) | 73.2 (71.5-74.8) | 31.3 (28.5-34.1) | 78.7 (77.1-80.2) | 1.42 (1.28-1.57) | 0.85 (0.80-0.89) |

ISS, Injury Severity Score; NFTI, Need for Trauma Intervention; HR, heart rate; RR, respiratory rate; SBP, systolic blood pressure; CI, confidence interval

**Supplementary Table 6.** Comparison of empirically derived vital sign criteria, the PALS criteria, and the ATLS criteria for detecting major trauma in patients aged < 14.

| **Vital sign criteria** | **Sensitivity, % (95% CI)** | **Specificity, % (95% CI)** | **PPV, % (95% CI)** | **NPV, % (95% CI)** | **PLR (95% CI)** | **NLR (95% CI)** |
| --- | --- | --- | --- | --- | --- | --- |
| **PALS** | | | | | | |
| HR | 48.6 (41.1-56.2) | 75.8 (73.8-77.7) | 16.0 (13.0-19.3) | 94.0 (92.7-95.1) | 2.01 (1.69-2.38) | 0.68 (0.59-0.78) |
| RR | 55.2 (47.7-62.6) | 60.8 (58.5-63.0) | 11.8 (9.7-14.2) | 93.4 (91.9-94.8) | 1.41 (1.22-1.62) | 0.74 (0.602-0.87) |
| SBP | 66.5 (59.1-73.3) | 41.5 (39.3-43.7) | 9.7 (8.1-11.5) | 92.9 (91.0-94.5) | 1.14 (1.02-1.27) | 0.81 (0.65-1.00) |
| **ATLS** | | | | | | |
| HR | 35.4 (28.4-42.8) | 83.6 (81.9-85.3) | 17.1 (13.4-21.3) | 93.1 (91.8-94.3) | 2.16 (1.73-2.70) | 0.77 (0.69-0.86) |
| RR | 11.0 (6.9-16.5) | 91.7 (90.4-92.9) | 11.3 (7.0-16.9) | 91.5 (90.2-92.8) | 1.34 (0.86-2.08) | 0.97 (0.92-1.02) |
| SBP | 6.6 (3.5-11.3) | 98.9 (98.3-99.3) | 36.4 (20.4-54.9) | 91.8 (90.5-92.9) | 6.00 (3.00-12.00) | 0.94 (0.91-0.98) |
| **Empirically derived using simplified age-based cutoff** | | | | | | |
| HR | 45.3 (37.8-52.8) | 79.3 (77.4-81.1) | 17.1 (13.8-20.8) | 93.9 (92.6-95.0) | 2.18 (1.82-2.62) | 0.69 (0.60-0.79) |
| RR | 46.9 (39.4-54.5) | 66.6 (64.5-68.8) | 11.7 (9.5-14.3) | 93.0 (91.5-94.3) | 1.41 (1.19-1.67) | 0.80 (0.69-0.92) |
| SBP | 44.7 (37.3-52.3) | 74.7 (72.7-76.7) | 14.3 (11.5-17.5) | 93.5 (92.1-94.7) | 1.77 (1.48-2.12) | 0.74 (0.65-0.85) |

ISS, Injury Severity Score; NFTI, Need for Trauma Intervention; HR, heart rate; RR, respiratory rate; SBP, systolic blood pressure
